# Improved Training and Semen Collection Outcomes Using the Closed Box Chair for Macaques

**DOI:** 10.3390/ani11082384

**Published:** 2021-08-12

**Authors:** Lisa A. Houser, Cathy Ramsey, Fernanda M. de Carvalho, Breanna Kolwitz, Chelsey Naito, Kristine Coleman, Carol B. Hanna

**Affiliations:** 1 Oregon National Primate Research Center, Division of Comparative Medicine, Oregon Health & Science University, Beaverton, OR 97006, USA; houserl@ohsu.edu (L.A.H.); kolwitz@ohsu.edu (B.K.); 2 Oregon National Primate Research Center, Division of Reproductive and Developmental Sciences, Oregon Health & Science University, Beaverton, OR 97006, USA; ramseyc@ohsu.edu (C.R.); decarval@ohsu.edu (F.M.d.C.); chelsey.naito@gmail.com (C.N.); hannaca@ohsu.edu (C.B.H.)

**Keywords:** restraint, refinement, semen volume, sperm concentration, stress, welfare

## Abstract

**Simple Summary:**

Refining procedures is an important part of promoting animal welfare. One area in which refinements are particularly important is animal restraint. Monkeys and other animals may be restrained for a variety of reasons, including sperm collection. In this study, we compared the use of a closed box chair (CBC) as a potential refinement over the more traditional open restraint chair (ORC) for restraint. We trained 34 male rhesus macaques to enter either the CBC or the ORC, allow restraint, and provide a semen sample. While all monkeys were reliably trained for the CBC, only 75% completed the task in the ORC. Further, it took longer to train the monkeys for the ORC than the CBC. Importantly, monkeys restrained in the CBC produced a higher ejaculatory volume and a higher sperm concentration than those in the ORC. Taken together, these data suggest that the CBC reduces stress for the animals while improving scientific outcomes, and thus is a refinement over the ORC.

**Abstract:**

Collaborative semen collection in monkeys is a valuable tool in research, animal collection management, and conservation efforts. To obtain samples, monkeys are often restrained in open restraint chairs (ORC) with the “pole and collar” technique. While commonly used, this restraint is not tolerated by all individuals; some become anxious or aggressive towards the poles and people. In an effort to refine this procedure and improve welfare of the monkeys, we examined the use of a “closed box chair” (CBC), a clear, plexiglass box in which the monkey is trained to sit for sperm collection. The CBC does not require pole and collar, and although legs are secured, the arms and neck are not restrained. The use of CBCs has increased in recent years; however, there are few studies demonstrating its effects on scientific outcomes. We used positive reinforcement techniques to train 34 adult male rhesus macaques (*Macaca mulatta*) to provide semen samples using either the ORC or the CBC. While all CBC monkeys (*n* = 14) were reliably trained for this procedure, only 75% of ORC (*n* = 20) males completed the training (*p* = 0.04). It took significantly less time to train animals in the CBC than the ORC (201.0 vs. 412.4 min; *p* <0.001). In a controlled subset, males restrained with ORC (*n* = 7) produced a significantly lower ejaculatory volume than those collected by CBC (*n* = 10) (297.6 µL vs. 522.1 µL respectively; *p* = 0.04) and had a lower concentration of sperm (186.0 × 10^6^/mL vs. 367.5 × 10^6^/mL respectively; *p* = 0.017), although there were no differences with respect to sperm motility (*p* = 0.15). Our data suggest the closed box chair technique reduces stress on the animals while enhancing semen quality, supporting the use of the CBC as an important refinement.

## 1. Introduction

The past decade has seen a dramatic shift in refinements to research and husbandry practices. These refinements are often focused on reducing stress for the animals while improving scientific outcomes. One procedure known to affect stress in nonhuman primates (NHPs) is restraint. Restraint activates the hypothalamic pituitary adrenal (HPA) axis in NHPs and other species [1,2,3,4,5,6] which, in turn, can negatively impact research outcomes.

Because of the associated stress, there has been a concerted effort to refine the use of restraint. The use of restraint is identified in both the Animal Welfare Act [7] and the *Guide for the Care and Use of Laboratory Animals* [8] as something that should be used only when necessary for achieving research goals and for the least amount of time possible. Further, there has been a great deal of effort focused on ways to reduce the need for restraint. In many situations, restraint can be reduced through the use of positive reinforcement training [9]. For example, monkeys can be trained to put their arm in a blood sleeve attached to their cage for blood draw [10] thus reducing the need for overall restraint.

However, there are situations in which restraint is still used for laboratory NHPs, such as when animals are being asked to remain in one location for extended periods of time (e.g., electrophysiological recordings of neurons are taken while animals are engaged in behavioral testing) or for procedures which may be challenging to perform in the home cage (e.g., for some biological sampling). In these situations, animals are often restrained with a primate chair. While there are a variety of styles of primate chairs, they can be broken down into two main types: the open restraint chair (ORC) and the closed box chair (CBC). The “original” chair design is open [11] and allows access to all of the animal’s body. This design utilizes a “pole and collar” technique to move the monkey from the home cage into the chair. The monkey is first outfitted with an aluminum “collar” which goes around the neck. One or two poles can then be attached to the collar to guide the monkey from the cage to the chair. Once in the chair, the collar is fitted into a neck yoke and the monkey’s arms or legs are restrained as necessary depending on what body part is to be accessed. In recent years, there has been a concerted effort to increase the amount of training provided to the animals who undergo this process. Positive reinforcement training is used to provide animals with opportunities to cooperate with this procedure. Still, even with training, this kind of restraint is not tolerated by all individuals; some become anxious or aggressive towards the poles and people. There are also concerns using this method in regards to safety for both animals and technicians [12]. Further, use of pole and collar is not considered best practice in Europe [13].

An alternative to the open restraint chair is the closed box chair (CBC; [14,15]), an enclosed acrylic box, most frequently designed with an opening at the top that allows the monkey to lift his head through. There is often a yoke that is fitted around the neck and it may have additional restraints built into it, depending on its use. In many cases, this design allows the monkey to move directly from its home cage into the chair. It does not require the use of pole and collar, although that is still utilized in some situations. See McMillan et al. 2017 for more details [16].

There is a great deal of overlap between the kinds of studies that employ these two styles of chairs. The CBC is often used for neuroscience studies, as it provides access to the monkey’s head while securing the rest of the body, while the ORC is more likely to be used for biological sample collection [16]. However, this distinction is often based on the facility and the kind of equipment available. There are few studies directly comparing the two methods of restraint. Further, there are few studies demonstrating the effects of either kind of restraint on scientific as well as welfare outcomes.

Reproductive science is a major research focus at the Oregon National Primate Research Center (Beaverton, OR, USA). As such, there are many research protocols that require semen collection from macaques for various reasons, including for use in assisted reproductive technologies. Semen can be collected from sedated animals by using either penile stimulation or a rectal probe to facilitate sample collection [17,18,19,20,21]. However, we have observed a higher failure rate of obtaining semen samples from sedated animals by penile stimulation (personal observation) compared to “awake” collections. Further, it has been reported that coagulum recovered from rectal probe collections in Japanese macaques and lowland gorillas often fail to liquefy and motile sperm recovery is not possible, making collections without sedation preferable. There are some examples to date of Great Apes being trained to use an artificial vagina for semen collection [22,23]; however, this kind of training has not proven effective in macaques [24]. Therefore, we currently use non-sedated, penile electroejaculation for routine semen collection.

The semen collection procedure itself is relatively quick (approximately 30–120 s); however, it does require the animal to remain still, and thus a primate chair is often utilized. Animals are typically removed from their cage, secured in the chair, and then wheeled to the room in which the procedure will take place. We used the ORC for this procedure for many years. However, as others have found [14], we noticed that monkeys were not always successfully trained for this procedure. Further, monkeys that we were able to train to calmly accept the chair restraint often continued to appear fearful of and/or aggressive to the pole, which has been reported elsewhere [16]. Lack of fearful behavior does not mean that the procedure is not stressful; monkeys that appear to have acclimated to the chair behaviorally can still display physiological indicators of stress [25]. Thus, in an effort to refine our practices, we modified a CBC to be used for semen collection.

As with the introduction of any new animal procedure, it is important to survey for all potential welfare, staff safety, and scientific data collection concerns and challenges. Protocols that increase dedicated staff time or impact scientific outcomes are typically challenging to implement. Therefore, to determine whether the CBC is a refinement over the ORC for semen collection, we performed a retrospective study to evaluate the amount of time it took to train naïve monkeys to enter and allow restraint in the two types of chairs. Importantly, we also compared standard quality metrics from semen collected from a subset of these animals using the two different restraint methods as physiological stress can induce oxidative stress, negatively impacting male fertility [26,27,28,29]; thus, semen quality metrics can be used as a biomarker for the animals’ level of comfort with each restraint system. 

## 2. Materials and Methods

### 2.1. Subjects

The subjects for this study were 34 adult (5–14 year old) male rhesus macaques (*Macaca mulatta*). All subjects were born at the Oregon Primate National Research Center (ONPRC; Beaverton, OR, USA). They were assigned to various research protocols in which collecting semen was needed (e.g., male contraception, fertility preservation, infertility, propagation of specific genotypes, and zygote gene editing). Because it is well established that temperament can influence collaboration and training success in rhesus macaques [30,31], in many cases, we assessed the monkeys prior to selecting them as subjects for these studies. However, in some cases (*n* = 4 for both chair types), monkeys were selected based on particular characteristics. For example, individuals genotyped as heterozygous carriers for specific disease-associated mutations, such as BBS7^+/−^, were selected as subjects because collection of their gametes was essential to the development of biomedical model cohorts.

Thirty of the subjects were mother-reared in large outdoor groups (consisting of between 25 and 200 individuals) and 4 of the subjects were peer-reared in a nursery [32]. Subjects had been living indoors for at least 1 month prior to the start of training, although most had been indoors for more than a year (average +/− SD = 1038 +/− 614 days). The monkeys were housed in standard monkey cages sized in accordance with the *Guide for the Care and Use of Laboratory Animals* [8], in animal rooms that contained 12–32 monkeys. Sixteen subjects were single housed and 10 were paired with a social partner for the duration of training. The remaining 8 males were pair housed part of the time (from 4–30+ days; see Table S1 for specific details). Monkeys were fed standard monkey chow (Purina LabDiet Chow, St. Louis, MO, USA) twice a day, and were given fresh produce or other food enrichment daily. Water was provided freely through automatic lixit systems. The lights were on 12 h per day, from 0700 to 1900, and the temperature maintained at 24 ± 2 °C. Subjects participated in the ONPRC behavioral management program to ensure their psychological health and well-being. The ONPRC animal care program is compliant with the Animal Welfare Act Regulations [7] and accredited by AAALAC, International. The ONPRC Institutional Care and Use Committee approved all studies in which these animals participated.

### 2.2. Training

**Figure 1:**
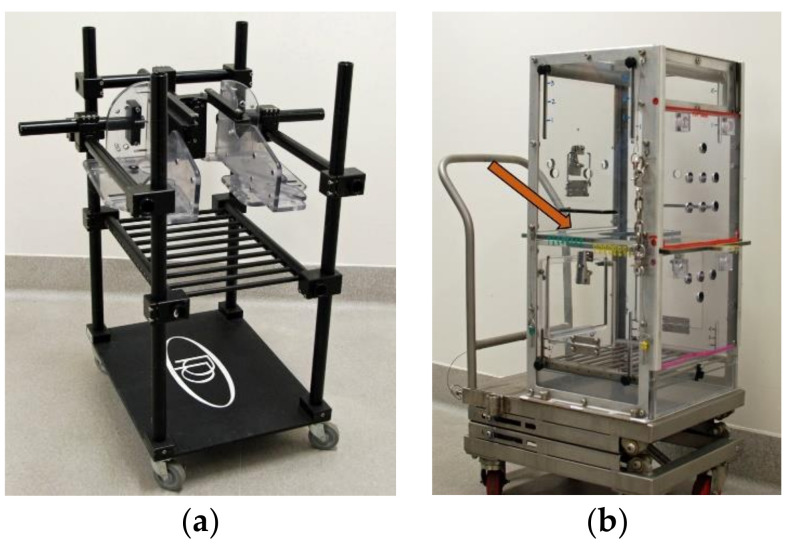
The open restraint chair (ORC; **a**) and closed box chair (CBC; **b**). Arrow indicates waist plate modification.

The training goal for all subjects was to enter a restraint chair and allow a semen sample to be collected via electroejaculation. Twenty subjects underwent training to enter a standard open-style restraint chair (ORC; Primate Products, Immokalee, FL, USA; Figure 1a) using the “pole and collar” technique to move from their home cage into the chair. Prior to training, these monkeys were outfitted with a commercially available aluminum primate collar (Primate Products, Immokalee, FL, USA). The remaining 14 subjects were trained to enter a closed-style box chair (CBC; Carter2Systems, Hillsboro, OR, USA; Figure 1b) directly from their home cage. The CBC used in this study was a custom design that eliminated the neck and arm restraint, and instead utilized waist plates to allow safe access to the lower half of the monkey’s body. All animals were naïve to training. The approach to training for both chairs followed a method similar to that described in McMillan et al. 2014 [33] and used primarily positive reinforcement, counterconditioning, and desensitization techniques. See Supplementary Tables S2 and S3 for abbreviated training plans. Pair housed animals were temporarily separated from their partner with a mesh slide for the duration of the training session, after which the partners were promptly reunited. While we utilized positive reinforcement techniques whenever possible, negative reinforcement (i.e., using the cage’s squeeze back to bring monkeys closer to the front of the cage) was employed as necessary to complete training during the timeframe provided (approximately 6–8 weeks).

Training sessions for both chairs were conducted 5 days per week and varied considerably in length (from 3–35 min) depending on the goals for the session (e.g., to attach and then release the pole to the collar 3 times, shut the door to the box two times). Training was considered complete once monkeys calmly entered the chair, allowed the necessary restraints to be put into place (arms and legs secured in the ORC; waist plates inserted and legs secured in the CBC), and the first semen sample was collected. If an animal became overly aggressive to the trainers or exhibited elevated levels of anxiety (e.g., excessive teeth grinding, self-directed aggression, etc.) over the course of several sessions, training was discontinued entirely. All training sessions were conducted by two technicians, which included at least one senior, highly experienced trainer. Training for both methods was coordinated by the ONPRC Training Specialist, who developed the shaping plans, ensured consistency of approach amongst all of the trainers, and monitored the progress of the training for each monkey.

### 2.3. Semen Collection

To examine the effect of restraint type on sperm quality, we evaluated samples collected from a subset of subjects (*n*_ORC_ = 10, *n*_CBC_ = 7) who underwent routine sample collections (see Table S1 for details on which subjects were included). All semen collections were performed by electroejaculation without sedation using a PTE 110 Volt AC electroejaculator (P-T Electronics, Model 303, Boring, OR, USA) as previously described [24]. Prior to placement of the defibrillation strips, the penis was examined for wounds and tissue inconsistencies that would disqualify the animal from the procedure. Gentle pressure was used to extend the penis to apply electrode cream and wrap defibrillation strips cut to size at the base and behind the glans. A 10–15 s priming stimulus was applied followed by a slow and steady increase of voltage to produce a slight erection, engorgement of the glans, or elevation of the testicles into the inguinal region. Engorgement and ejaculation were achieved between 10–20 V (not to exceed the safety cut-off limit of 35 V) within 90–120 s. of stimulation. If unsuccessful after the first attempt, the procedure could be repeated twice, but only if the animal remained collaborative of the process. Ejaculate was collected directly into a wide mouth container and allowed to sit at 37 °C for 30 min. to allow for the liquefaction of the sperm rich fraction from the coagulum.

### 2.4. Sperm Isolation and Analysis

The liquid portion of the ejaculate was measured by pipetting to obtain the total volume, then transferred to a 15 mL conical tube. The coagulated plug was washed with 3 mL of TALP-HEPES (Tyrode’s Albumin, Lactate, Pyruvate with HEPES buffer, made in house) supplemented with 3 mg/mL of bovine serum albumin [34] to recover remaining spermatozoa then combined with the liquid fraction before adding additional TALP-HEPES Q.S. to 12 mL. Samples were washed by centrifugation at 300× *g* for 7 min at 37 °C to pellet the spermatozoa. Supernatant was aspirated and pellet resuspended in fresh TALP-HEPES Q.S. to 12 mL before a second centrifugation for a final wash. After removal of the supernatant (11 mL), the sperm pellet was resuspended in the remaining 1 mL of TALP-HEPES and analyzed by a computer assisted sperm analysis (CASA) system (IVOS II-Animal Motility software, version 1.11, Hamilton Thorne, Beverly, MA, USA) programmed specifically for macaques. This was used to determine sperm motility and concentration for samples diluted 1:20 in TALP-HEPES. The settings for the CASA were as follows: frame rate 60 Hz, frames acquired 100, minimum tail brightness 79, elongation 1–100, head size 9–60 µm^2^, progressive average path velocity (VAP) threshold 25 µm/s, progressive straightness (STR) threshold 80%, slow VAP cutoff 20 µm/s, slow straight-line velocity (VSL) cutoff 10 µm/s, static VAP cutoff 0 µm/s, static VSL cutoff 0 µm/s, static width multiplier 0.5.

### 2.5. Data Analysis

#### 2.5.1. Training Data

For each subject, we calculated the amount of time (in minutes) and the number of sessions that it took to reliably perform the task (i.e., enter chair, sit calmly, and provide semen sample). If training was ended due to behavioral reasons, training was considered unsuccessful. To examine whether there was a difference in training time between the two methods, we compared both the number of sessions and the total time (minutes) for the animals who successfully completed their training. 

#### 2.5.2. Semen Quality Comparison

We examined the quality of semen from the subset of 17 subjects for whom we had baseline sample collections. Our goal was to use data from 10 baseline collections for these monkeys; however, we were only able to get 7–9 samples for 4 of the animals due to project requirements. Therefore, we included the 7–10 consecutive collections immediately following completion of training in the comparison for a total of 177 samples used in this study (73 ORC and 104 CBC). Semen samples were collected over a single breeding season for each monkey and recorded for liquid volume (µL), sperm concentration (1 × 10^6^ sperm/mL), and total sperm motility (%). Values for quality parameters were averaged for each male before combining to determine the overall results for the ORC and CBC methods.

#### 2.5.3. Statistical Analysis

Data are presented as the average value +/− standard error of the mean (SEM). We used chi square analysis to determine if the percentage of animals successfully completing this training differed based on technique. For all other variables (training and semen quality), we tested normality using the Shapiro–Wilk test. We then compared data from animals tested in the ORC and the CBC using a two-tailed Student’s *t*-test or Mann–Whitney U test (when transformations did not normalize the data). Alpha values were set at 0.05. SYSTAT 11 (Systat Software Inc., San Jose, CA, USA) was used to analyze data.

## 3. Results

### 3.1. Training

A significantly higher percentage of animals were successfully trained for the closed box chair compared to the open restraint chair (chi square = 4.103, df = 1, *p* = 0.04). We were able to train all 14 monkeys to enter the CBC, allow restraints to be applied, and allow semen collection using predominantly positive reinforcement techniques. In contrast, only 15 of 20 males (75%) were successfully trained to enter the ORC and allow semen collection. In general, training was discontinued because the animals showed excessive aggression towards the trainer or stress towards the procedures during multiple sessions. Of the remaining 15 ORC animals, 2 required extra restraints during semen collection and 2 additional animals were eventually released from the project due to behavioral reasons. One animal trained with the CBC was released from his protocol for behavioral reasons and one had underlying health issues causing poor semen quality. 

**Figure 2:**
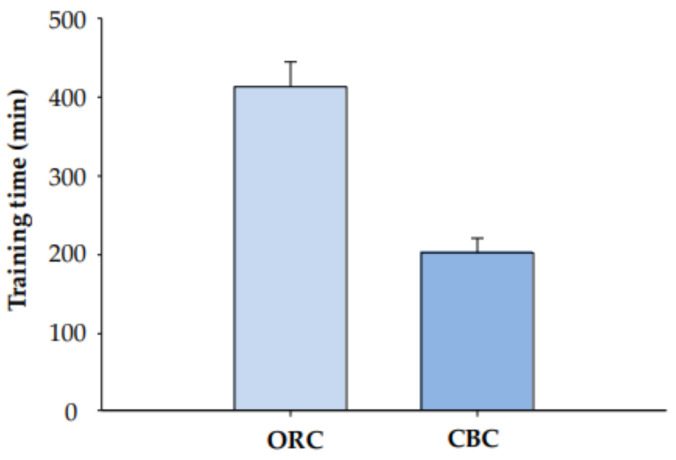
Amount of time (in minutes) it took to train monkeys to allow semen collection in either the ORC (*n* = 15) or CBC (*n* = 14).

There was considerable variation in the amount of time it took the 29 animals who were successfully trained (*n*_CBC_ = 14, *n*_ORC_ = 15). Some individuals learned the task relatively quickly, while others took significantly longer. There was no difference in the number of training sessions (Mann–Whitney U = 93, *p* = 0.61) between the two methods (25.9 +/− 1.3 sessions vs. 29.1 +/− 2.4 sessions for CBC vs. ORC monkeys, respectively). However, it took significantly less time (in minutes) to train animals for the CBC than ORC (*t* = 5.703, df = 27, *p* < 0.001; Figure 2). Trainers spent about twice as many minutes training monkeys for the ORC (412.4 +/− 31.7 min) than CBC (201.0 +/− 17.8 min).

### 3.2. Semen Quality Comparison

**Figure 3:**
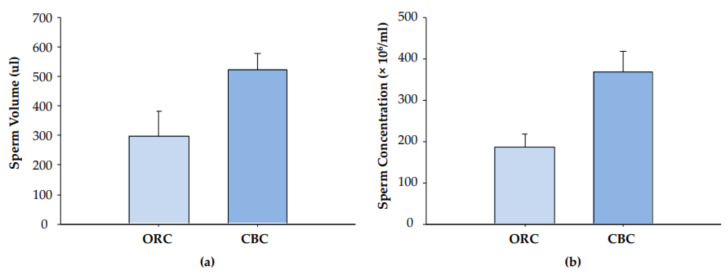
Sperm volume (**a**) and concentration (**b**) from monkeys restrained in either the ORC (*n* = 7) or the CBC (*n* = 10).

Overall, semen samples collected from males in the CBC system had a greater volume of 522.1 +/− 56.4 µL compared to those from ORC restrained animals (297.6 +/− 83.8 µL; t = 2.32, df = 15, *p* = 0.04; Figure 3a), with samples ranging from 340–940 µL (CBC) and 40–360 µL (ORC). A significant difference was also observed in the sperm concentrations; 367.5 +/− 51.1 × 10^6^/mL and 186.0 +/− 32.3 × 10^6^/mL for CBC and ORC, respectively (*t* = 2.70, df = 15, *p* = 0.017; Figure 3b). No changes were observed between the restraint systems in sperm motility with typical values expected from reproductive age males collected by both methods; 74.7 +/− 8.3 vs. 88.6 +/− 1.9% for ORC and CBC, respectively (Mann–Whitney U = 49.5, *p* = 0.154).

## 4. Discussion

Collaborative semen collection in monkeys is a valuable tool in research, animal collection management, and conservation efforts. The “traditional” method of restraining monkeys for this procedure has been with an open restraint chair (ORC). However, not all animals tolerate restraint in this chair well; some monkeys become anxious and/or aggressive to the procedure, and may need to be removed from the project. We modified a closed box chair (CBC), often used for restraint in neuroscience research, for use with semen collection, and evaluated its use with male rhesus macaques. Our data suggest that, compared to the ORC, the CBC reduces stress on the animals while enhancing semen quality, and as such, is an important refinement.

This study was retrospective, and thus we did not directly quantify behavior of the animals or take cortisol samples before and after restraint. However, monkeys were more compliant with training in the CBC than in the ORC, which suggests that they found it less aversive. While all of the animals trained in the CBC successfully met training criteria (i.e., enter, sit calmly, allow semen collection), a quarter of animals trained in the ORC could not complete the training due to behavioral issues such as aggression or anxiety. These monkeys were ultimately removed from the protocol, resulting in additional animals needing to undergo the training. Further, it took significantly less time (in minutes) to train monkeys in the CBC than in the ORC, due largely to the increased cooperation of the monkeys. This increased cooperation lasted beyond the training itself. Monkeys entered the CBC more readily and seemed calmer and more relaxed in it than in the ORC. They also exhibited less aggression to staff and showed fewer behavioral indices of stress (e.g., teeth-grinding) in the CBC than the ORC (personal observation).

Importantly, the quality of the semen characteristics from monkeys restrained in the CBC was vastly improved over samples taken from monkeys restrained in the ORC, both in terms of volume and concentration. Stress is known to negatively impact semen volume as well as sperm concentration and morphology in otherwise clinically healthy men [35,36,37]; thus, the enhanced quality suggests that the CBC was less aversive to the monkeys. It should be noted that concentration values are dependent on the recovery of sufficient sample volume and could be underrepresented as a result of partial ejaculatory success. For instances where there were very small sample volumes recorded (e.g., >10 µL), particularly as within the ORC cohort, incomplete emission likely occurred so that the spermatocyte complement of the total sample was artificially low and not necessarily an indicator of changes in gametogenesis. However, these low concentration values demonstrate the importance of being able to reliably collect from donor males. This is particularly important in fertility and toxicology studies where repeated collections and measurement of changes to semen characteristics is a data collection endpoint and consistency in collection protocols is key. Clearly, using a restraint system that minimizes stress is critical for maintaining consistency and ensuring samples which are as accurate as possible. We were able to reduce stress and thereby improve the semen quality and collection reliability with the CBC apparatus which, in turn, improves the quality of research being conducted and increases the likelihood of recovering valuable genetics. Interestingly, there was no difference in the motility of the sperm between animals restrained in the two systems. The increased quality of the semen samples suggests that monkeys may need to provide fewer samples and thus undergo the collection procedure fewer times with the CBC than the ORC, which is a clear refinement. Further, reducing the need for samples could result in a reduction in animals used, another of the 3Rs [38].

In addition to reducing stress for the NHPs, the CBC has also been reported as less stressful for the humans [16]. Staff at our facility who were involved in procedures using both chair types reported a preference for the CBC over the ORC. Similarly, we have found that the CBC is safer for both humans and NHPs. There were several events in which technicians received injuries as a result of using the pole and collar. In contrast, we have had no such events since switching to the closed box chair. This is due, in part, to the enclosed structure of the chair; there is a barrier between the hands and mouths of the NHPs and the technicians. Further, there are fewer chances for mechanical and/or human error, such as a collar breaking or a pole slipping off of the collar while a monkey is out of its cage. However, the increased safety is likely also due to the behavior of the monkeys themselves. As mentioned above, the monkeys were calmer in the CBC than the ORC, and working with a calm, cooperative monkey is safer than working with an anxious monkey [39].

There are several reasons why restraint in the CBC may be less stressful than in the ORC. First, as mentioned above, the CBC does not rely on the pole and collar. In an international survey on the use of chair restraint, respondents reported that they felt the pole and collar was among the most challenging steps in the chairing process [16]. This result is not surprising for anyone who has performed this procedure. The poles attach to a collar which sits on the animal’s neck, and thus come very close to the animal’s face. There is little room for error on the part of the trainer. The loss of control a monkey may experience while being guided out of its cage on the pole can also be stressful. While many animals eventually acclimate to this process, some do not. Further, the ORC requires more manipulation than the CBC; monkeys are removed from their cage and either walked to or manually placed in the chair. In contrast, most CBCs are designed so that monkeys can move directly from their cage to the chair, providing them with greater control over their environment, which has been shown to reduce stress [40,41]. Additionally, our version of the CBC has neither a neck yoke nor arm restraints, which affords the monkeys significantly more freedom of movement; even with the waist plate and leg restraints in place, the animals are contained, rather than restrained. The CBC also allows for a more natural sitting posture for the collection procedure than the ORC, which likely increases comfort for NHPs.

While it took less time in minutes for monkeys trained in the CBC to reach training criteria than those trained in the ORC, there was no difference in the number of training sessions. This finding is likely due to the designated time frame provided to the trainers. Trainers had approximately 6 weeks to train monkeys prior to the start of the protocol. For all training, regardless of the type of chair, we tried to use positive reinforcement training (PRT) whenever possible. However, given the time constraint, if animals had not reached a particular threshold by a certain time point (e.g., allowing the pole to be attached to the collar after 10 sessions), we implemented a degree of negative reinforcement training (NRT). For example, we used the squeeze back of the cage to encourage monkeys to move towards the cage door. This combination of primarily PRT with some NRT is relatively common in restraint training [14,33,42]. 

The CBC is often used for neuroscience studies and in this study we utilized it for semen collection. However, the CBC can be used for other purposes as well. We have trained several male rhesus macaques to cooperate with unsedated blood collections from either the saphenous or femoral veins in the CBC for pharmacokinetic studies. In addition, we are currently training female rhesus macaques to enter the chair and remain stationary for ultrasounds. In both of these examples, utilizing the CBC reduces the need for sedation. As anesthetic agents such as Ketamine HCl have been correlated with increased cortisol levels and decreases in appetite after recovery [43] in macaques, reducing their use is also a refinement.

Overall, we have demonstrated the ability to improve animal welfare in male rhesus macaques by changing the type of restraint system from the traditional open restraint chair to the closed box chair. Furthermore, we have established improved semen collection outcomes as a result of decreased stress in the animals which provides significant value to genetic banking and assisted reproductive technology programs.

## 5. Conclusions

Our findings demonstrate that the closed box chair (CBC) is a refinement over the more traditional open restraint chair (ORC) for semen collection in rhesus macaques. Monkeys were more likely to be successfully trained to enter the chair, allow restraint, and provide a semen sample in the CBC than the ORC. It took less time to train monkeys for the CBC than for the ORC, due largely to the increased cooperation of the subjects. This increased compliance persisted past the training period; monkeys restrained with the CBC entered the chair more readily, remained calmer, and exhibited less aggression to staff, than those restrained with the ORC. Importantly, monkeys restrained in the CBC produced a higher ejaculatory volume and sperm concentration than those in the ORC, suggesting that the CBC was less aversive. Taken together, these findings demonstrate that, compared to the ORC, the CBC reduces stress for monkeys thereby improving the semen quality and collection reliability which, in turn, enhances the quality of research being conducted.

## Data Availability

The data presented in this study are available on request from the corresponding author.

## Supplementary Materials

The following are available online at https://www.mdpi.com/article/10.3390/ani11082384/s1. Table S1: Demographic information, training outcome (whether or not successfully trained), and whether or not their semen data were included, for study subjects. Social housing status refers to the predominant manner in which the monkey was housed (single housed or paired) during the training sessions; animals that changed status were identified as paired/single, Table S2: Abbreviated shaping plan used to train animals to enter the open restraint chair and allow semen collection, Table S3: Abbreviated shaping plan used to train animals to enter the closed box chair and allow semen collection.
