# Supplementary material for: Improved Training and Semen Collection Outcomes Using the Closed Box Chair for Macaques
Source: Animals (Basel). 2021 Aug 12;11(8):2384. doi: 10.3390/ani11082384 (PMC8388729; doi:10.3390/ani11082384)
Supplement: Supplementary file 1 [file animals-11-02384-s001.zip › animals-1319023-supplementary.pdf]

animals

MDPI

Supplementary Materials

Improved Training and Semen Collection Outcomes Using the Closed Box Chair for Macaques

Lisa A. Houser [1], Cathy Ramsey [2], Fernanda M. de Carvalho [2], Breanna Kolwitz [1], Chelsey Naito [2], Kristine Coleman [1,*] and Carol B. Hanna [2]

[1] Division of Comparative Medicine, Oregon National Primate Research Center, Oregon Health & Science University, Beaverton, OR 97006, USA; houserl@ohsu.edu (L.A.H.); kolwitz@ohsu.edu (B.K.)
[2] Division of Reproductive and Developmental Sciences, Oregon National Primate Research Center, Oregon Health & Science University, Beaverton, OR 97006, USA; ramseyc@ohsu.edu (C.R.); decarval@ohsu.edu (F.M.d.C.); naito@gmail.com (C.N.); hannaca@ohsu.edu (C.B.H.)
* Correspondence: colemank@ohsu.edu

Citation: Houser, L.A.; Ramsey C.; Carvalho F.M.d.; Kolwitz B.; Naito C.; Coleman K.; Hanna C.B. Improved Training and Semen Collection Outcomes Using the Closed Box Chair for Macaques. *Animals* **2021**, *11*, 2384. https://doi.org/10.3390/ani11082384

Academic Editor(s): Mark J. Prescott; Melanie L. Graham

Received: 14 July 2021
Accepted: 9 August 2021
Published: 12 August 2021

Publisher's Note: MDPI stays neutral with regard to jurisdictional claims in published maps and institutional affiliations.

Copyright: by the authors. Licensee MDPI, Basel, Switzerland. This article is an open access article distributed under the terms and conditions of the Creative Commons Attribution (CC BY) license (http://creativecommons.org/licenses/by/4.0/).

Copyright: © 2021 by the authors. Licensee MDPI, Basel, Switzerland. This article is an open access article distributed under the terms and conditions of the Creative Commons Attribution (CC BY) license (http://creativecommons.org/licenses/by/4.0/).

Table S1. Demographic information, training outcome (whether or not successfully trained), and whether or not their semen data were included, for study subjects. Social housing status refers to the predominant manner in which the monkey was housed (single housed or paired) during the training sessions; animals that changed status were identified as paired/single.

Subject	Chair type	Age at start of training	Social housing status during training	Rearing history	Trained successfully	Included in semen analysis
C1	CBC	12	Single	Mother	Y	N
C2	CBC	12	Single	Mother	Y	Y
C3	CBC	14	Paired/single	Mother	Y	Y
C4	CBC	10	Paired	Mother	Y	Y
C5	CBC	9	Single	Mother	Y	N
C6	CBC	7	Single	Mother	Y	N
C7	CBC	7	Paired/single	Mother	Y	Y
C8	CBC	8	Paired	Mother	Y	Y
C9	CBC	8	Paired	Mother	Y	Y
C10	CBC	8	Paired	Mother	Y	Y
C11	CBC	7	Paired	Mother	Y	Y
C12	CBC	6	Paired	Mother	Y	Y
C13	CBC	5	Paired/single	Mother	Y	Y
C14	CBC	5	Paired	Mother	Y	N
O1	ORC	11	Single	Mother	N	N
O2	ORC	11	Single	Mother	N	N
O3	ORC	10	Single	Mother	N	N
O4	ORC	8	Paired	Mother	Y	N
O5	ORC	10	Paired/single	Mother	Y	N
O6	ORC	8	Single	Mother	N	N
O7	ORC	8	Single	Mother	Y	N
O8	ORC	10	Single	Mother	Y	N
O9	ORC	7	Single	Mother	Y	Y
O10	ORC	8	Single	Mother	Y	Y
O11	ORC	6	Paired	Mother	Y	Y
O12	ORC	5	Paired/single	Nursery	Y	Y
O13	ORC	5	Paired/single	Nursery	Y	Y
O14	ORC	5	Single	Nursery	Y	N
O15	ORC	7	Single	Mother	Y	N
O16	ORC	7	Single	Mother	Y	N
O17	ORC	5	Paired/single	Mother	Y	Y
O18	ORC	7	Single	Mother	Y	N
O19	ORC	5	Paired	Nursery	N	N
O20	ORC	5	Paired/single	Mother	Y	Y

Table S2. Abbreviated shaping plan used to train animals to enter the open restraint chair and allow semen collection.

**Description of training steps for open restraint chair (ORC)**
Use counterconditioning techniques to desensitize subject to the movement/use of the squeeze back restraint mechanism in the home cage. This step is undertaken to reduce stress associated with this procedure in case it is needed during the course of training
Habituate animals to presence of equipment (e.g., chair, poles) while conducting the next 5 steps of training
Use shaping techniques to train subject to sit on the floor of the home cage in front of the cage door
Reinforce subject for remaining seated while pole is inserted into the cage and slowly moved toward the subject
Desensitize subject to the touch of the pole on a neutral body part (such as the leg or back) and gradually work up towards attaching the pole to the collar
Once the subject allows the first pole to be attached, reinforce him for sitting calmly while the cage door is opened slightly, allowing the second pole to be attached to the opposite side of the collar
Fully open the cage door and encourage the subject to exit his cage and walk on floor by placing reinforcement small distances away from the cage opening
Once subject is comfortable walking with the poles attached to the collar, gently guide him to the ORC and allow him to climb onto the platform
Gently guide the collar into the neck yoke, secure it in place and then remove the poles
Desensitize the subject to manipulation and restraint of the arms and legs
Desensitize the subject to genital manipulation and to the equipment to be used for semen collection

Table S3. Abbreviated shaping plan used to train animals to enter the closed box chair and allow semen collection.

**Description of training steps for closed box chair (CBC)**
Use counterconditioning techniques to desensitize subject to the movement/use of the squeeze back restraint mechanism in the home cage. This step is undertaken to reduce stress associated with this procedure in case it is needed during the course of training
Place CBC in front of subject's home cage and use shaping techniques to train subject to enter chair and allow doors to be closed
Use shaping techniques to train subject to sit on the floor of the chair
Reinforce subject for remaining seated while plates are inserted and secured around the waist
Desensitize subject to to manipulation and restraint of the legs
Desensitize the subject to genital manipulation and to the equipment to be used for semen collection
